# Adding Psychotherapy to the Naltrexone Treatment of Alcohol Use Disorder: Meta-analytic Review

**DOI:** 10.7759/cureus.3107

**Published:** 2018-08-06

**Authors:** Rizwan Ahmed, Vijaya Padma Kotapati, Ali M Khan, Nuzhat Hussain, Mudasar Hussain, Sara Dar, Jeevan Kumar, Gulshan A Begum, Michael Esang, Navjot Brainch, Saeed Ahmed

**Affiliations:** 1 Psychiatry, Liaquat College of Medicine & Dentistry, Karachi, PAK; 2 Psychiatry, Manhattan Psychiatric Center, New York, USA; 3 Psychiatry Resident, University of Texas Rio Grande Valley, Harlingen, Texas, USA; 4 Psychiatry, Penn State University College of Medicine, Pennsylvania, USA; 5 Psychiatry, NYU Langone Medical Center, New York, USA; 6 Psychiatry, Brigham and Women's Hospital, Boston, USA; 7 Psychiatry, Bolan Medical College, Quetta, PAK; 8 Behavioral Health Sciences, Nassau University Medical Center, East Meadow, USA; 9 Psychiatry, Maimonides Medical Center, Brooklyn, USA

**Keywords:** alcohol use disorder, naltrexone, psychotherapy, abstinence, relapse, gamma-glutamyl transferase, cravings

## Abstract

Background

It remains unclear if naltrexone combined with psychotherapy is superior to naltrexone alone in treating alcohol use disorders (AUD). The current meta-analysis examined the hypothesis that psychotherapy is a significant moderator that influences AUD-related outcomes and that naltrexone combined with psychotherapy is associated with significantly better AUD-related outcomes than naltrexone alone.

Methods

A total of 30 studies (N_naltrexone _= 2317; N_placebo _= 2056) were included. Random effects model meta-analyses were carried out for each of the studied outcomes. Subsequently, the random effects model pooled estimates from studies with and without psychotherapy were compared using a Wald test. A mixed-effect model, incorporating psychotherapy as a moderator, was used to examine the impact of psychotherapy on treatment outcomes.

Results

Naltrexone had a significant treatment effect on abstinence relapse and Gamma-Glutamyl Transferase levels, but not cravings. The pooled estimates for studies with and without psychotherapy were not significantly different for any of the studied outcomes. Psychotherapy was not a significant moderator in the mixed effects models for any of the studied outcomes.

Conclusions

Naltrexone treatment is efficacious in reducing alcohol consumption, but not reducing cravings. Adding psychotherapy on top naltrexone did not result in any significant additional benefit for AUD patients.

## Introduction

Alcohol use disorders (AUD) are debilitating psychiatric illnesses characterized by the abuse of and dependence on alcohol. The lifetime prevalence of AUD is estimated to be at 29.1%. AUD bring about devastating consequences to the individual. They are associated with a high disease-related burden – chronic alcohol consumption is causally implicated in cancer, cardiovascular disease, liver cirrhosis, and injury. On top of that, an estimated 3.8% of deaths and 4.6% of disability life years are attributable to alcohol. At the societal level, the economic costs associated with AUD amounts to at least 1% of the gross national product in high and middle-income countries. Given such consequences, the need to treat AUD cannot be understated. Ever since naltrexone was approved as a treatment for alcohol dependence in 1994 by the U.S. Federal Drug Administration, it has been widely used as a first-line pharmacological treatment for AUD. As a μ-opioid receptor antagonist, naltrexone blocks alcohol-related dopamine release in the mid-brain reward system, thereby reducing the reward and reinforcement associated with alcohol consumption.

Many controlled trials have been carried out over the past three decades to examine the efficacy of naltrexone on various AUD-related outcomes and several meta-analyses have been carried out to synthesize the results of these studies. These meta-analyses have generally indicated moderate to strong efficacy in treating AUD with naltrexone. However, these meta-analyses included studies that carried out various psychotherapies to augment the naltrexone treatment of AUD. Two studies [1, 2] had previously examined the effect of adding psychotherapy to naltrexone treatment in treating AUD, within-study, and concluded that adding psychotherapy to the naltrexone treatment of AUD did not result in significantly better outcomes. Apart from these two studies, it remains largely unclear if the significant treatment effect is primarily attributed to naltrexone alone or the combination of naltrexone and psychotherapy. Such information would be useful to optimize existing naltrexone interventions on AUD.

To this end, in addition to examining the overall efficacy of naltrexone on AUD, we also examined the impact of psychotherapy in two different ways. First, we compared the pooled estimates between studies with and without psychotherapy; then we examined the moderation effect of psychotherapy on the treatment outcomes. As with previous meta-analyses on AUD intervention studies, we hypothesized that the meta-analysis would yield significant effect sizes favoring the use of naltrexone on AUD. Additionally, based on a previous meta-analysis comparing between pharmacological-only treatment and combined psychotherapy and pharmacological treatment, we hypothesized that studies of naltrexone treatment combined with psychotherapy yield significantly better AUD-related outcomes relative to those of naltrexone alone studies.

## Materials and methods

Data sources and extraction

Using the Preferred Reporting Items for Systematic Reviews and Meta-Analyses (PRISMA) methodology, a search for relevant published literature was carried out on PubMed, Medline and Cochrane using the keywords “naltrexone”, “low dose naltrexone”, “high dose naltrexone”, “naltrexone alcohol”, “naltrexone psychotherapy”. This search started on 2nd February 2017 and was last carried out on 30th March 2017. The reference lists of relevant studies were also manually searched for additional studies. Only articles in English were retrieved. We included studies of controlled trials involving naltrexone treatment on AUD. Specifically, these studies must be of an independent group's pretest-posttest design. There were no restrictions on patients’ age or level of alcohol use. Researches that studied AUD-related outcomes using experimental/laboratory paradigms were not included in the meta-analysis.

**Figure 1:**
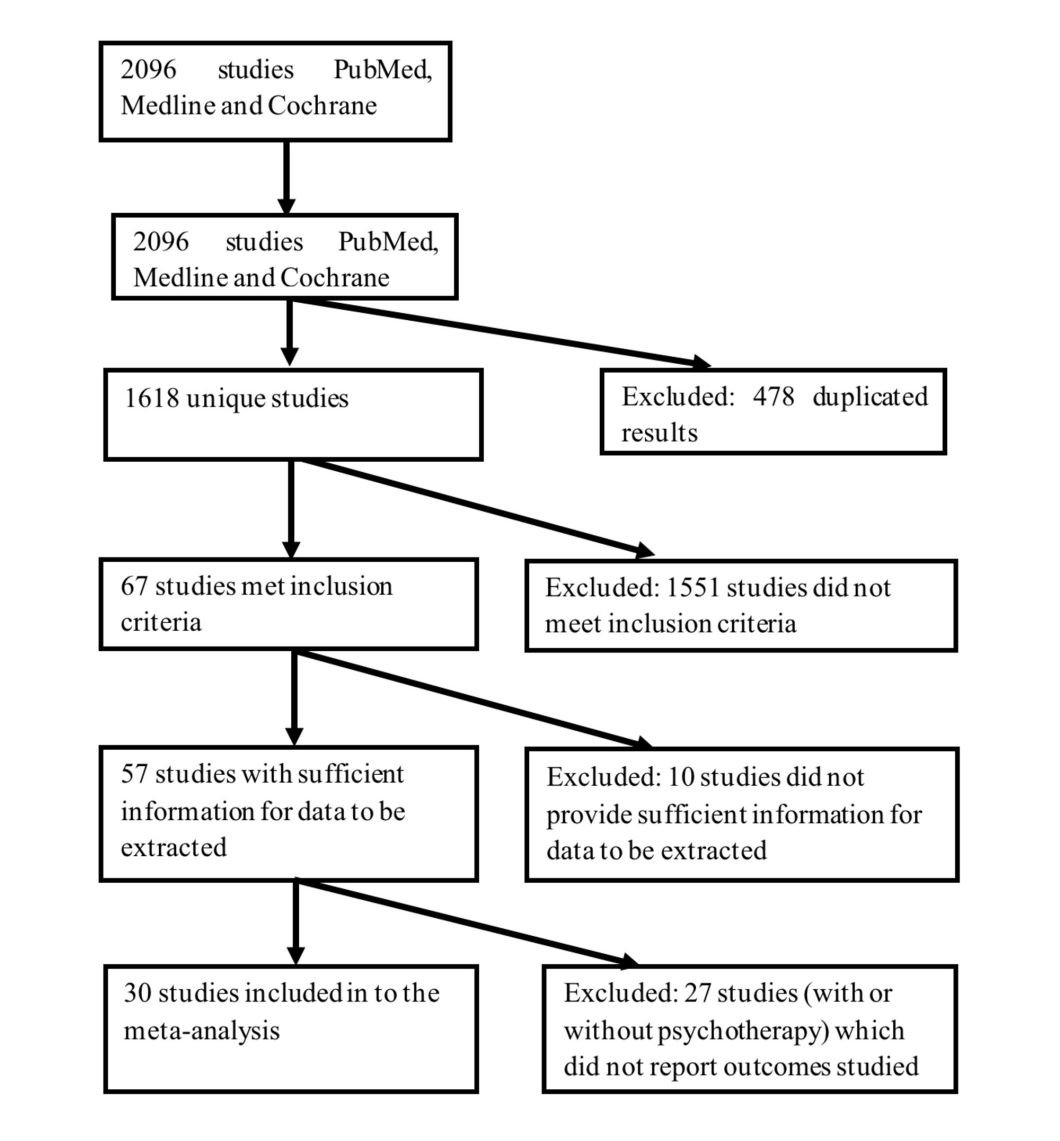
Selection of studies.

Means and standard deviations (SD) as well as event counts of the trial outcomes, and information pertaining to the participants (sample size, age, sex, diagnosis), and trial-design (naltrexone dose, duration and psychotherapy) for each study were entered into a structured data abstraction form by the two research assistants. If there were discrepancies among both of them, the entered data was checked against the source. In cases, where there were multiple naltrexone treatment groups (i.e., different dosages), these means, SDs and event counts of these groups were combined using the formulae for combining groups. Due to the multitude of outcomes studied among the retrieved studies, only data from outcomes, which have been reported by at least four studies in each of the with and without psychotherapy subgroups, were extracted. For the purpose of the current study, we define psychotherapy as non-pharmacological treatment, such as individual and group therapy, psychoeducation, counseling and skills training, of at least two sessions, aimed at modifying cognition and/or behaviors associated with alcohol consumption. Hence, interventions that focused solely on adherence or medication compliance are not considered as psychotherapy. The flow chart for the selection of studies and data extraction is shown in Figure 1.

Data Synthesis

For outcomes involving dichotomous variables only at post-treatment, such as the number of participants who relapsed to heavy drinking or remained abstinent, the log Odds Ratios (OR) and their respective sampling variance were calculated using the escalc function in the metafor package in R. As for continuous outcomes studied both at baseline and post-treatment such as Gamma-Glutamyl Transferase (GGT) levels and cravings, the raw score single group pretest-posttest standardized mean difference (SMD) for both the treatment and placebo groups was computed separately using Formula A1. Following which, the placebo group’s SMD was subtracted from that of the treatment group to obtain the overall SMD for a particular studied outcome in the study. The sampling variances of the treatment and placebo subgroups were then summed to obtain the overall sampling variance for that outcome in the study.

These SMDs and ORs were then pooled and meta-analyzed using random effects models (REM), to allow the effect sizes to vary across studies. The direction of the effect sizes was coded in a manner that larger positive effect sizes correspond to larger positive differences (i.e., treatment – placebo) in raw scores. The Wald test was used to compare the pooled estimates between studies with and without psychotherapy. Next, a mixed effects model was meta-analyzed with psychotherapy (coded dichotomously for presence or absence) included as a moderator. Heterogeneity was measured using the Q statistic; a significant Q statistic suggests that the variability among the effect sizes is larger than what is expected from subject sampling error alone. In addition, trim and fill analyses were carried out to assess whether publication bias had significantly influenced the aggregated effect sizes. Specifically, the trim and fill analyses determine whether there are missing effect sizes, if so impute the missing effect sizes, and then recalculate the aggregated effect sizes. Follow-up leave-one-out analyses were carried out to assess the robustness of the results. These analyses were carried out using the metafor package in R 3.4.0.

## Results

**Figure 2:**
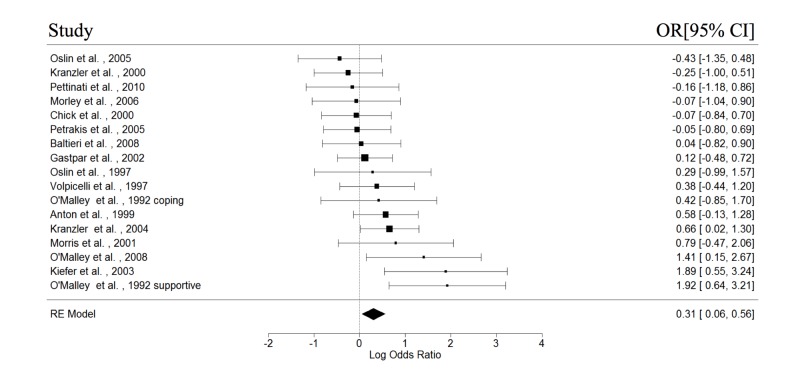
Forest plots of effect sizes for abstinence.

**Figure 3:**
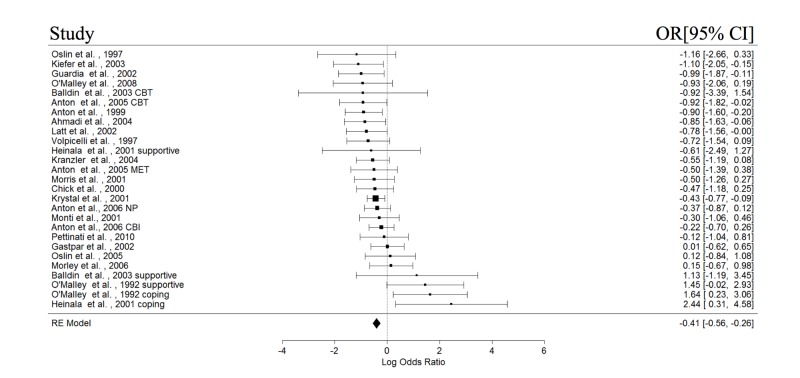
Forest plots of effect sizes for relapse.

**Figure 4:**
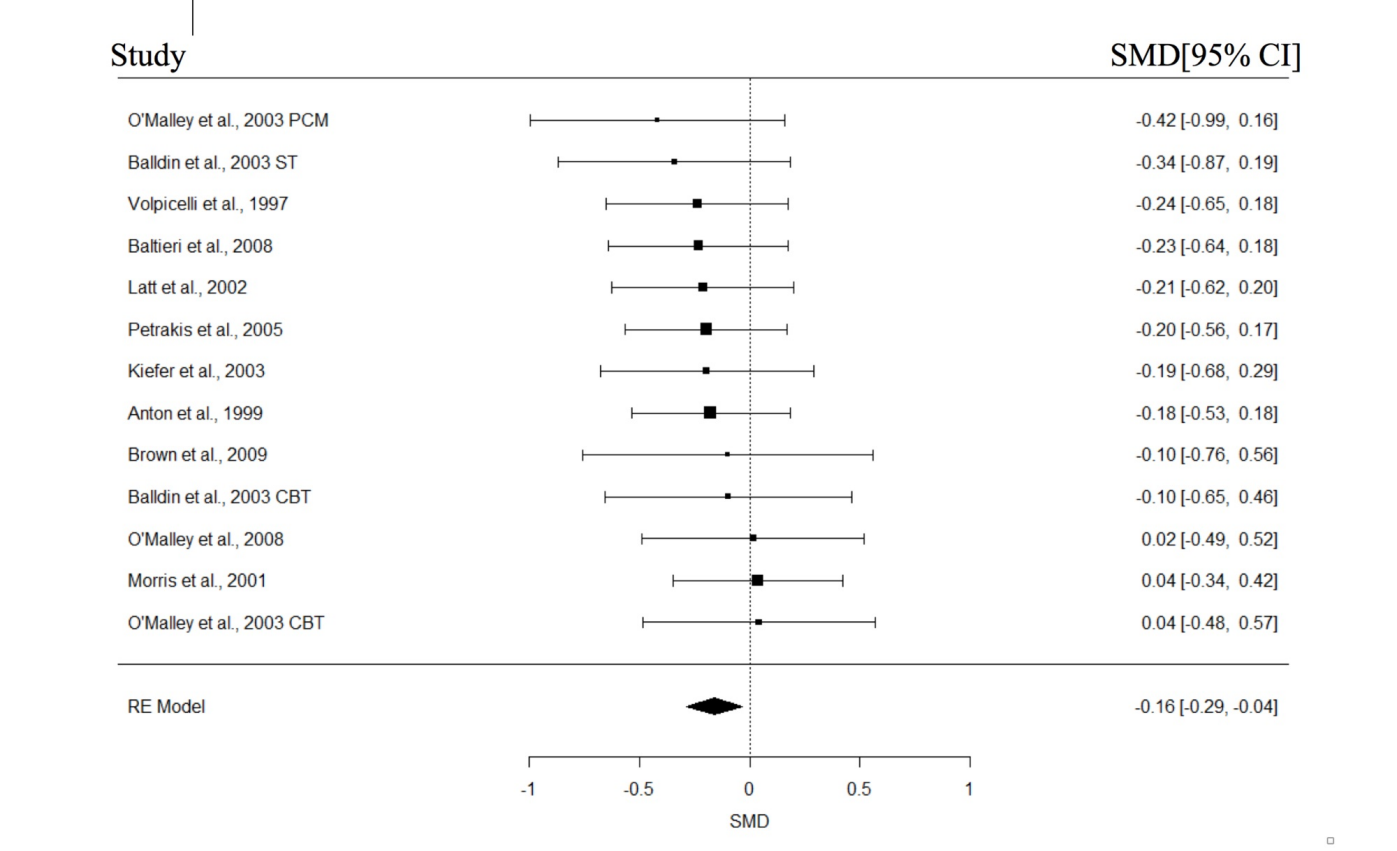
Forest plots of effect sizes for Gamma-Glutamyl Transferase.

**Figure 5:**
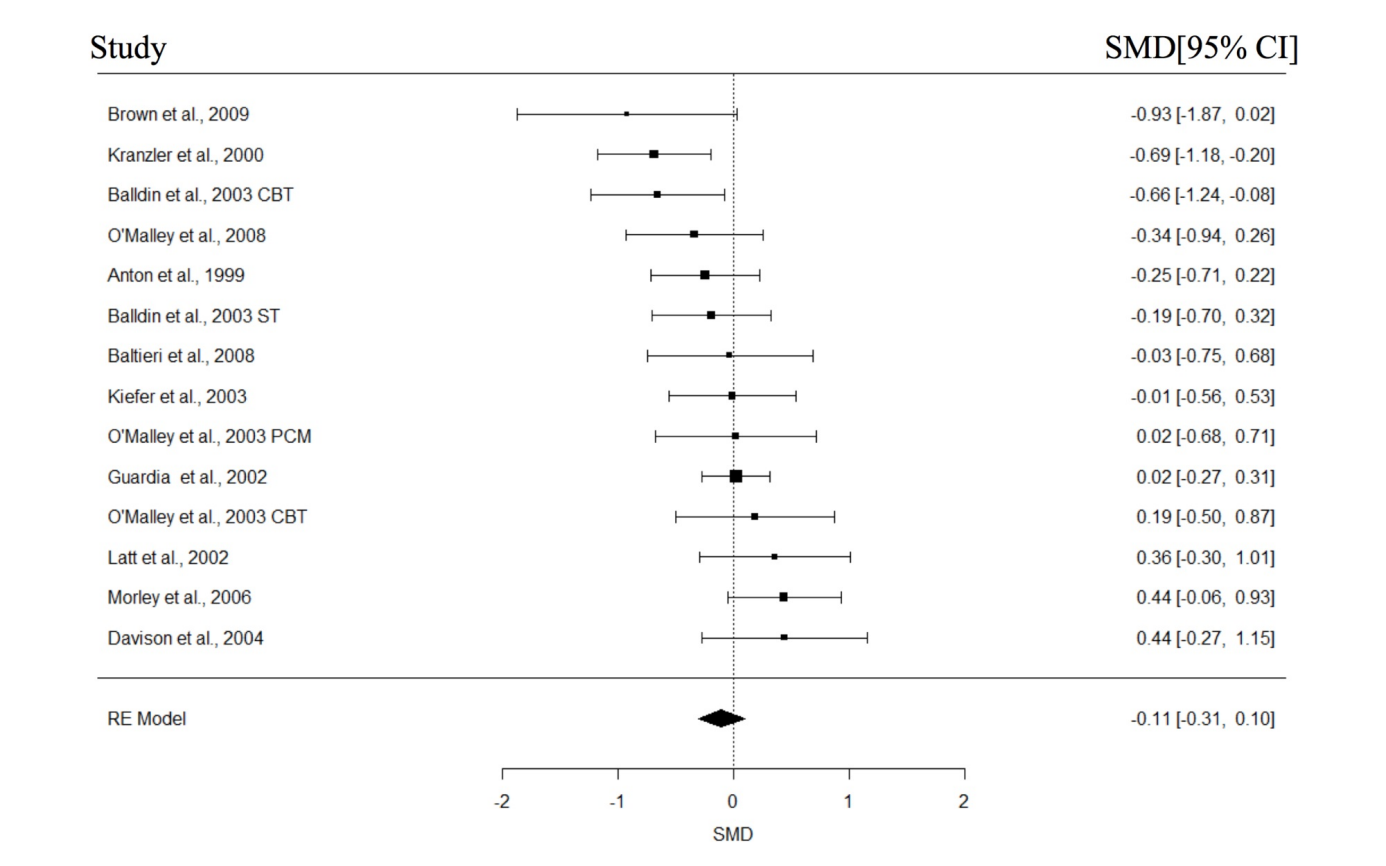
Forest plots of effect sizes for cravings.

The study and participant characteristics of all included studies are presented in Table 1 and Table 2, respectively. At least four studies in each of the with or without psychotherapy subgroups had reported data on abstinence, relapse, cravings, and GGT; these variables were then included in the meta-analyses. The results of the meta-analysis are shown in Table 3. The forest plots for the REMs of all studies are presented in Figures 2-5. Significant pooled estimates were obtained for the REM involving studies with psychotherapy for abstinence and relapse and all studies, for abstinence, relapse, and GGT. The significant negative effect sizes for relapse and GGT, and positive effect size for abstinence indicated that participants in the naltrexone treatment conditions had significantly better outcomes in these areas, relative to those in the placebo group. With the exception of a significant intercept in the mixed effect model (MEM) for relapse, none of the parameters in the MEMs was significant for any of the studied outcomes. Neither were the effect sizes in any of the craving meta-analyses statistically significant. Finally, none of the Wald test statistics was significant, suggesting that the pooled estimates of studies with and without psychotherapy were not significantly different.

**Table 1:** Study characteristics of included studies. d/d = drinks per day; d/o = drinks per occasion; dd/w = drinking days per week; BAC = Breath Alcohol Concentration; OCDS = Obsessive Compulsive Drinking scale; AUQ = Alcohol Urge Questionnaire; PACS = Penn Alcohol Craving Scale.

Study		Psychotherapy	Treatment dosage	Trial duration	Relapse definition	Measures of cravings
First author	Year					
Anton et al. [3]	2006	No psychotherapy	100 mg/day	16 weeks	≥4 drinks/day for women; ≥5 d/d for men	OCDS
Anton et al. [4]	2003	Cognitive behavioral therapy	50 mg/day	12 weeks		Analog scale
Anton et al. [3]	2006	Combined behavioral intervention	100 mg/day	16 weeks	≥4 d/d for women; ≥5 d/d for men	OCDS
O'Malley et al. [5]	1992	Coping skills	50 mg/day	12 weeks	≥4 d/o for women; ≥5 d/o for men	
O'Malley et al. [5]	1992	Supportive therapy	50 mg/day	12 weeks		
Oslin et al. [6]	1997	Group therapy	50 mg/day	12 weeks	≥ 5 d/occasion OR ≥ 5 dd/w or BAC > 100 mg/dl	
Petrakis et al. [7]	2005	No psychotherapy	50 mg/day	12 weeks		
Volpicelli et al. [8]	1997	Relapse prevention	50 mg/day	12 weeks	≥ 5 d/o or BAC >100 mg/dl	
Davidson et al. [9]	2004	Counseling	50 mg/day	10 weeks		
Kranzler et al. [10]	2000	Relapse prevention psychotherapy	50 mg/day	11 weeks		
Monti et al. [11]	2001	Coping skills training and communication skills training	50 mg/day	12 weeks	>5 d/d for women; >6 d/d for men	
O'Malley et al. [12]	2003	No psychotherapy	50 mg/day	24 weeks		OCDS
O'Malley et al. [12]	2003	Cognitive behavioral therapy	50 mg/day	24 weeks		OCDS
O'Malley et al. [13]	2008	No psychotherapy	1 x 12.5 mg + 2 x 25 mg + 50 mg/day thereafter	16 weeks	≥4 d/d for women; ≥5 d/d for men	AUQ
Oslin [14]	2005	No psychotherapy	50 mg/day	12 weeks	≥3 d/d for women; ≥4 d/d for men	
Pettinati et al. [15]	2006	Cognitive behavioral therapy	50 mg/day	11 weeks		
Brown et al. [16]	2009	Cognitive behavioral therapy	50 mg/day	12 weeks		PACS
Anton et al. [2]	2005	Cognitive behavioral therapy	50 mg/day	12 weeks	≥4 d/d for women; ≥5 d/d for men	
Anton et al. [2]	2005	Motivational enhancement therapy	50 mg/day	12 weeks	≥4 d/d for women; ≥5 d/d for men	
Kranzler et al. [17]	2004	Motivational enhancement therapy	300 mg + 3 months x 150 mg injection	12 weeks	≥4 d/d for women; ≥5 d/d for men	OCDS
Pettinati et al. [18]	2010	Cognitive behavioral therapy	100 mg/day	14 weeks	≥4 d/d for women; ≥5 d/d for men	
Baltieri et al. [19]	2008	Relapse prevention counseling	50 mg/day	12 weeks		OCDS
Guardia et al. [20]	2002	Supportive group therapy: relapse prevention + individual counseling	50 mg/day	12 weeks	>5 d/d for men; >4 d/d for women; or > 5dd/w	11-point Likert scale
Kiefer et al. [21]	2003	Group therapy: cognitive behavioral model of substance abuse	50 mg/day	12 weeks	≥5 d/d for men; ≥4 d/d for women; or ≥5 dd/w	OCDS
Morley et al. [22]	2006	No psychotherapy	50 mg/day	12 weeks	≥4 d/d for women; ≥6 d/d for men	PACS
Balldin et al. [23]	2003	Cognitive behavioral therapy	50 mg/day	6 months	≥4 d/d for women; ≥5 d/d for men	OCDS
Balldin et al. [23]	2003	Supportive therapy	50 mg/day	6 months	≥4 d/d for women; ≥5 d/d for men	OCDS
Chick et al. [24]	2000	Psychosocial treatment	50 mg/day	12 weeks	≥ 5 d/o for men; ≥ 4 d/o for women	
Heinala et al. [25]	2001	Coping (group therapy)	50 mg/day	12 weeks	≥ 5 d/o or ≥ 5 dd/w or intoxication at site visit	
Heinala et al. [25]	2001	Supportive therapy	50 mg/day	12 weeks	≥ 5 d/o OR ≥ 5 dd/w or intoxication at site visit	
Ahmadi et al. [1]	2004	Counseling	50 mg/day	36 weeks	≥ 5 d/o or ≥ 5 dd/w	
Gastpar et al. [26]	2002	Psychosocial treatment	50 mg/day	12 weeks	≥4 d/d for women; ≥5 d/d for men	
Morris et al. [27]	2001	Psychoeducation	50 mg/day	12 weeks	≥ 5 d/occasion or ≥ 5 dd/w or BAC > 100 mg/dl	
Latt et al. [28]	2002	Counseling and supportive therapy	50 mg/day	12 weeks	≥ 5 dd/w	OCDS
Toneatto et al. [29]	2009	Cognitive behavioral therapy	3 x 25 mg +11 x 50 mg	12 weeks		
Krystal et al. [30]	2001	Counseling	2 x 25 mg + 50 mg/day thereafter	3 months	≥4 d/d for women; ≥6 d/d for men	

**Table 2:** Participant characteristics of included studies. AUD = Alcohol use disorders; DSM = Diagnostic and Statistic Manual; SD = Standard Deviation; C = Coping; CBT = Cognitive Behavioral Therapy; ST = Supportive Therapy; NP = No psychotherapy; AD = Alcohol Dependence; AD/A = Alcohol dependence or abuse; AUDIT = Alcohol Use Disorder Inventory Test; MET = Motivational Enhancement Therapy; CBI = Combined Behavioral Intervention.

Study		AUD diagnosis	Number of participants (% male)				Age			
			Baseline		Follow Up		Treatment		Control	
First author	Year		Treatment	Placebo	Treatment	Placebo	Mean	SD	Mean	SD
Anton et al. [4]	2003	DSM-III-R AD	68 (69)	63 (73)	68	63	41	10	44	10
Anton et al. [3] (CBI)	2006a	DSM-IV AD	155 (68)	156 (71)	95	100	45.2	10.1	43.2	9.7
Anton et al. [3] (NP)	2006a	DSM-IV AD	154 (68)	103 (67)	96	89	44.4	9.9	44.2	9.2
O'Malley et al. [5] (C)	1992	DSM-III-R AD	29	25	19	15	42.8	10.3	38.5	8.8
O'Malley et al. [5] (ST)	1992	DSM-III-R AD	23	27	18	16				
Oslin et al. [6]	1997	DSM-III-R AD	21	23	14	13	56.5	6.8	58.9	6.7
Petrakis et al. [7]	2005	DSM-IV AD	59 (100)	64 (100)	46	40	47.7	7.4	46.2	7.3
Volpicelli et al. [8]	1997	DSM-III-R AD	48 (73)	49 (82)	35	36	39	9	37.9	8.5
Davidson et al. [9]	2004	AUDIT ≥ 8	19	19	16	19	46.5	10.5	50.8	7
Kranzler et al. [10]	2000	DSMIII-R AD	61 (80)	63 (75)			39.7	8.4	41.8	8.1
Monti et al. [11]	2001	DSM-IV AD/A	64	64						
O'Malley et al. [12] (NP)	2003	DSM-III-R AD	26	27	17	13				
O'Malley et al. [12] (CBT)	2003	DSM-III-R AD	30	30	19	24				
O'Malley et al. [13]	2008	DSM-IV AD	34 (65)	34 (62)	35	21	42	10.67	38.8	10.41
Oslin [14]	2005	DSM-IV AD	37 (78)	37 (81)			64.2	6.9	62.5	5.6
Pettinati et al. [15]	2006	DSM-IV AD	52 (75)	54 (70)	35	32	41.3	6.8	41.2	7.5
Brown et al. [16]	2009	DSM-IV AD	20 (50)	20 (45)	14	12				
Anton et al. [2] (CBT)	2005	DSM-IV AD	39 (79)	41 (73)	36	37	44	8	45	11
Anton et al. [2] (MET)	2005	DSM-IV AD	41 (73)	39 (77)	34	28	43	10	43	9
Kranzler et al. [17]	2004	DSM-IV AD	158 (67)	157 (63)	127	118	44.1	9.6	43.6	8.5
Pettinati et al. [18]	2010	DSM-IV AD	49 (67)	39 (56)	29	23	42.9	8.1	43.4	8.9
Baltieri et al. [19]	2008	ICD-10 AD	49 (100)	54 (100)	29	23	44.1	7.2	43.4	8.8
Guardia et al. [20]	2002	DSM-IV AD	101 (72)	101 (77)	61	59	41	8	42	9
Kiefer et al. [21]	2003	DSM-IV AD	40 (78)	40 (68)	22	10	46.1	8.1	45.6	11.1
Morley et al. [22]	2006	DSM-IV AD/A	53 (72)	61 (64)	36	40	47.6	8.5	42.4	9.3
Balldin et al. [23] (CBT)	2003	DSM-IV AD	25 (84)	30 (77)			50	7	50	8
Balldin et al. [23] (ST)	2003	DSM-IV AD	31 (87)	32 (91)			48	8	51	8
Chick et al. [24]	2000	DSM-III-R AD/A	90 (72)	85 (78)	37	36	43.1	8.3	43.9	9.7
Heinala et al. [25] (C)	2001	DSM-IV AD	34	33						
Heinala et al. [25] (ST)	2001	DSM-IV AD	29	25						
Ahmadi et al. [1]	2004	DSM-IV AD	58	58	26	15	42.76	9.58	43.19	8.81
Gastpar et al. [26]	2002	DSM-III-R AD/A	84 (77)	87 (68)	56	54	43.4	9.9	42	9.6
Morris et al. [27]	2001	DSM-III-R AD	55 (100)	56 (100)			47	8	48	8
Latt et al. [28]	2002	DSM-IV AD	56	51	38	36				
Toneatto et al. [29]	2009	DSM-IV AD/A	27	25	26	25				
Krystal et al. [30]	2001	DSM-IV AD	418 (97)	209 (98)	378	187	48.9	10	49.5	10

**Table 3:** Results of the meta-analyses. Note: Estimates for REM in the abstinence and relapse models are presented as odds ratios. K = Number of studies; CI = Confidence intervals; w/o = without; GGT = Gamma-Glutamyl Transferase; REM = Random Effects Model; MEM = Mixed Effects Model. *p < .05; **p < .01; ***p < .001.

Model/Parameter	K	N_treatment_	N_control_	Estimate	95% CI	Q	P_with vs. w/o psychotherapy_
Abstinence							
Random Effect Model (REM)	17	954	959	1.36*	1.06, 1.75	25.26	
With psychotherapy	13	771	763	1.45*	1.10, 1.91	22.01	.43
w/o psychotherapy	4	183	196	1.10	.59, 2.06	5.69	
Mixed Effect Model (MEM)	17	954	959			27.70	
Intercept				.07	-.47, .61		
Psychotherapy				.27	-.34, .88		
Relapse							
REM	27	1990	1725	.66***	.57, .77	40.33*	
With psychotherapy	23	1656	1434	.65***	.55, .77	35.59*	.67
w/o psychotherapy	4	334	286	.70*	-.68, .98	4.59	
MEM	27	1990	1725			41.80*	
Intercept				-.35*	-.69 -.02		
Psychotherapy				-.07	-.44, .30		
Gamma Glutamyl Transferase (GGT)							
REM	13	554	565	-.16*	-.29, -.04	3.74	
With psychotherapy	9	379	389	-.15	-.30, .01	2.37	.74
w/o psychotherapy	4	175	176	-.19	-.41, .03	1.26	
MEM	13	554	565			3.29	
Intercept				-.19	-.41, .03		
Psychotherapy				-.05	-.22, .32		
Cravings							
REM	14	626	640	-.11	-.31, .10	2.36	
With psychotherapy	10	457	467	-.19	-.42, .03	15.55	.14
w/o psychotherapy	4	169	173	.14	-.24, .51	4.42	
MEM	14	626	640			23.38*	
Intercept				.14	-.23, .51		
Psychotherapy				-.33	-.76, .10		

**Figure 6:**
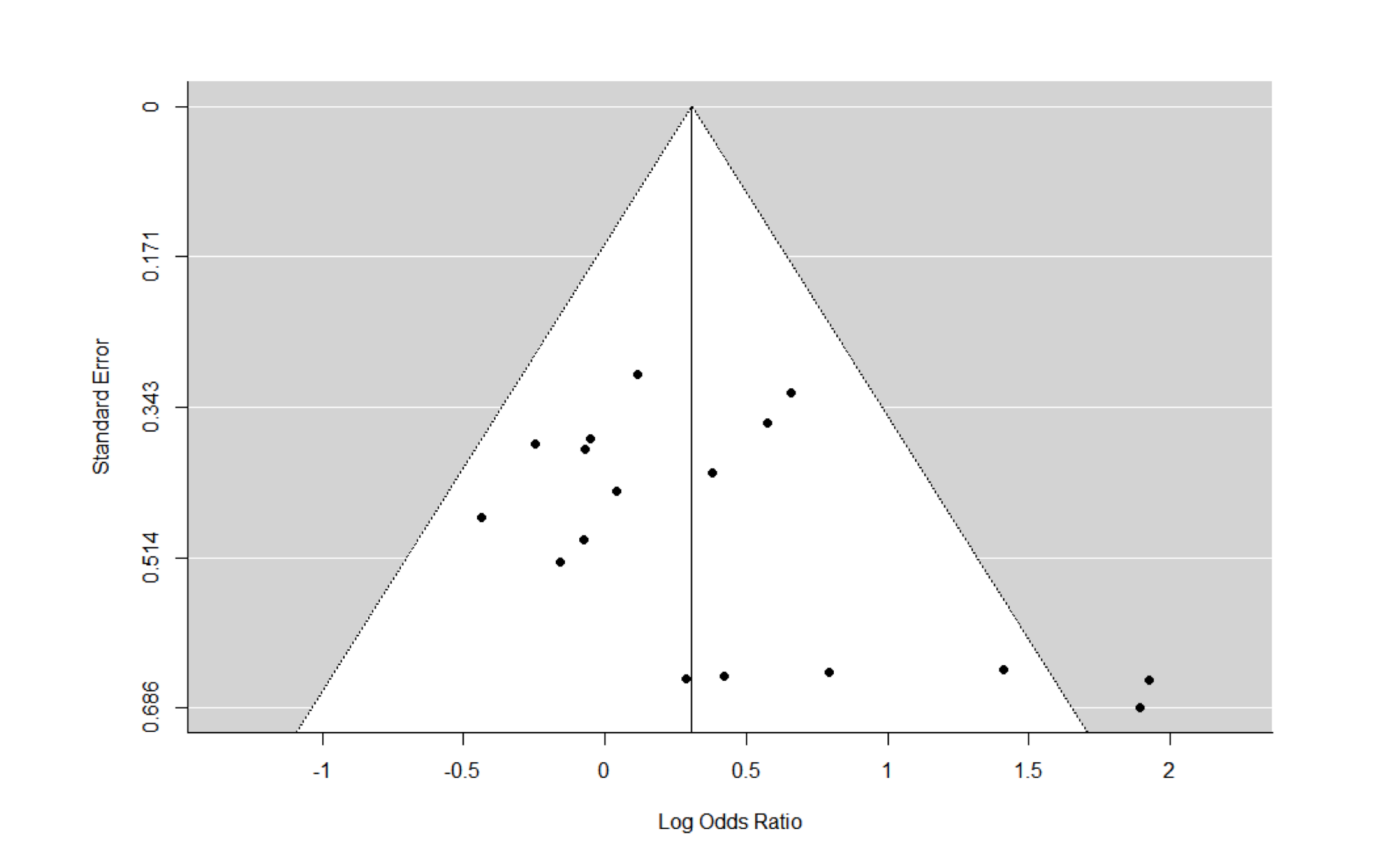
Funnel plots of standard errors plotted against effect sizes for identification of publication bias for abstinence.

**Figure 7:**
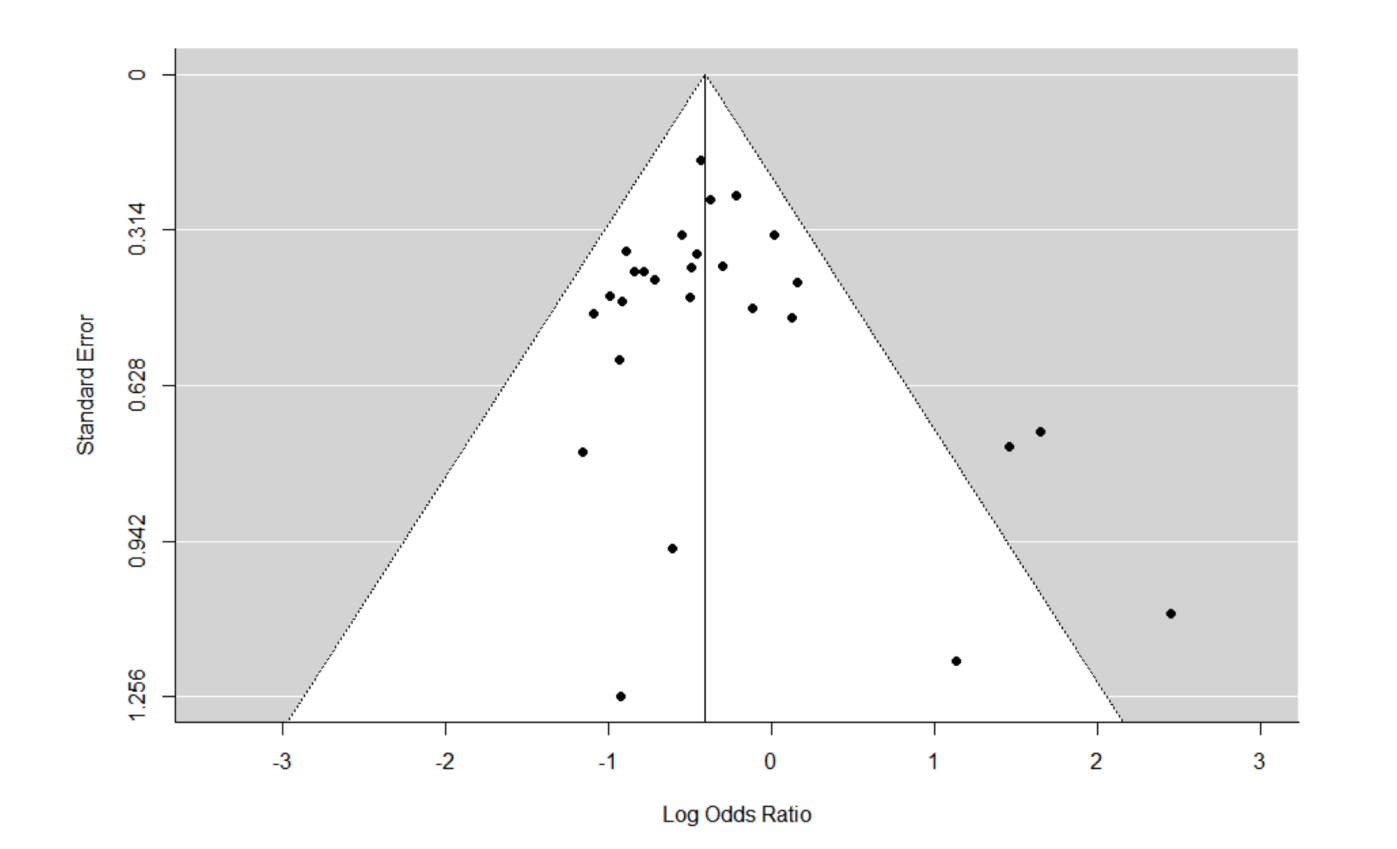
Funnel plots of standard errors plotted against effect sizes for identification of publication bias for relapse.

**Figure 8:**
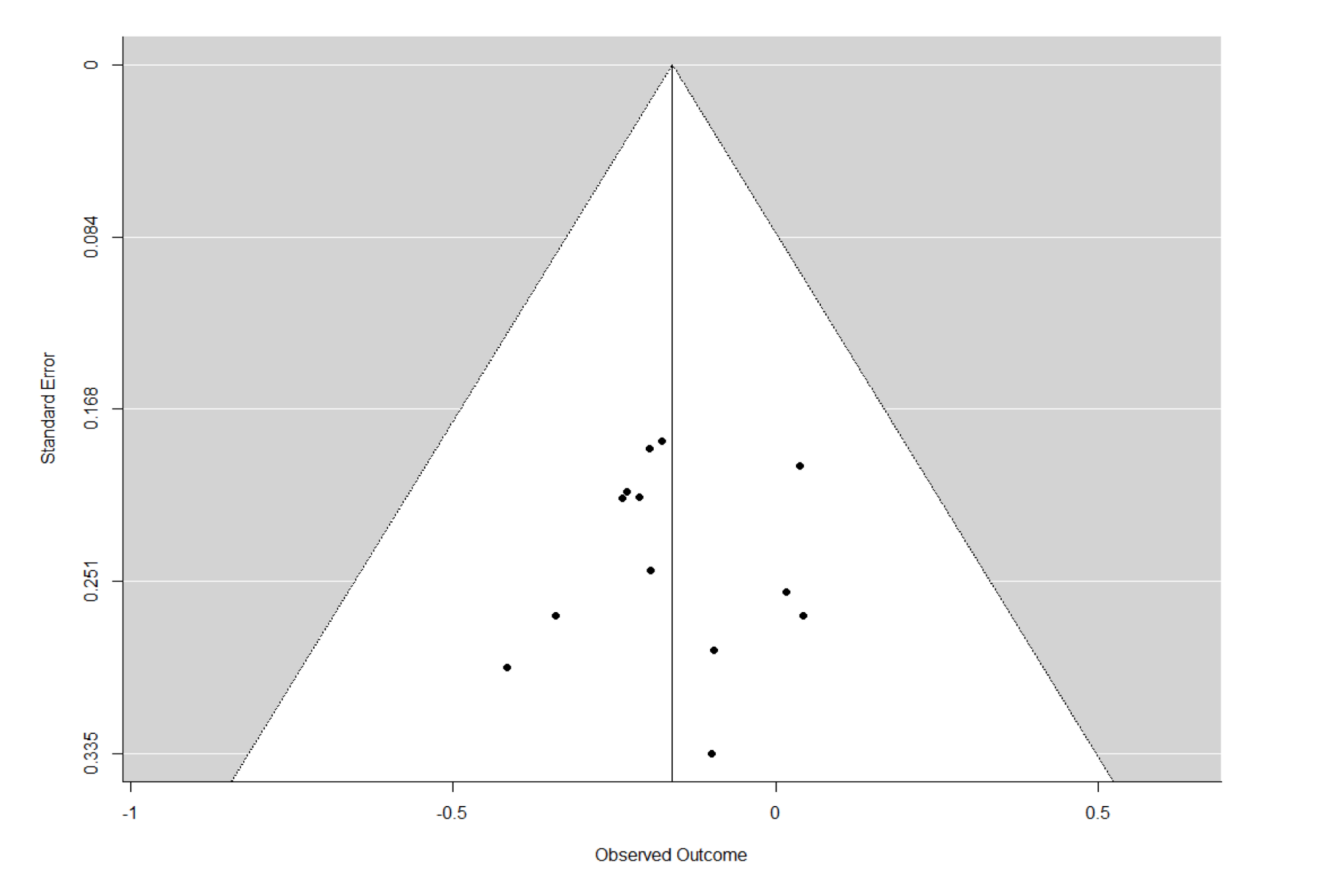
Funnel plots of standard errors plotted against effect sizes for identification of publication bias for Gamma-Glutamyl Transferase.

**Figure 9:**
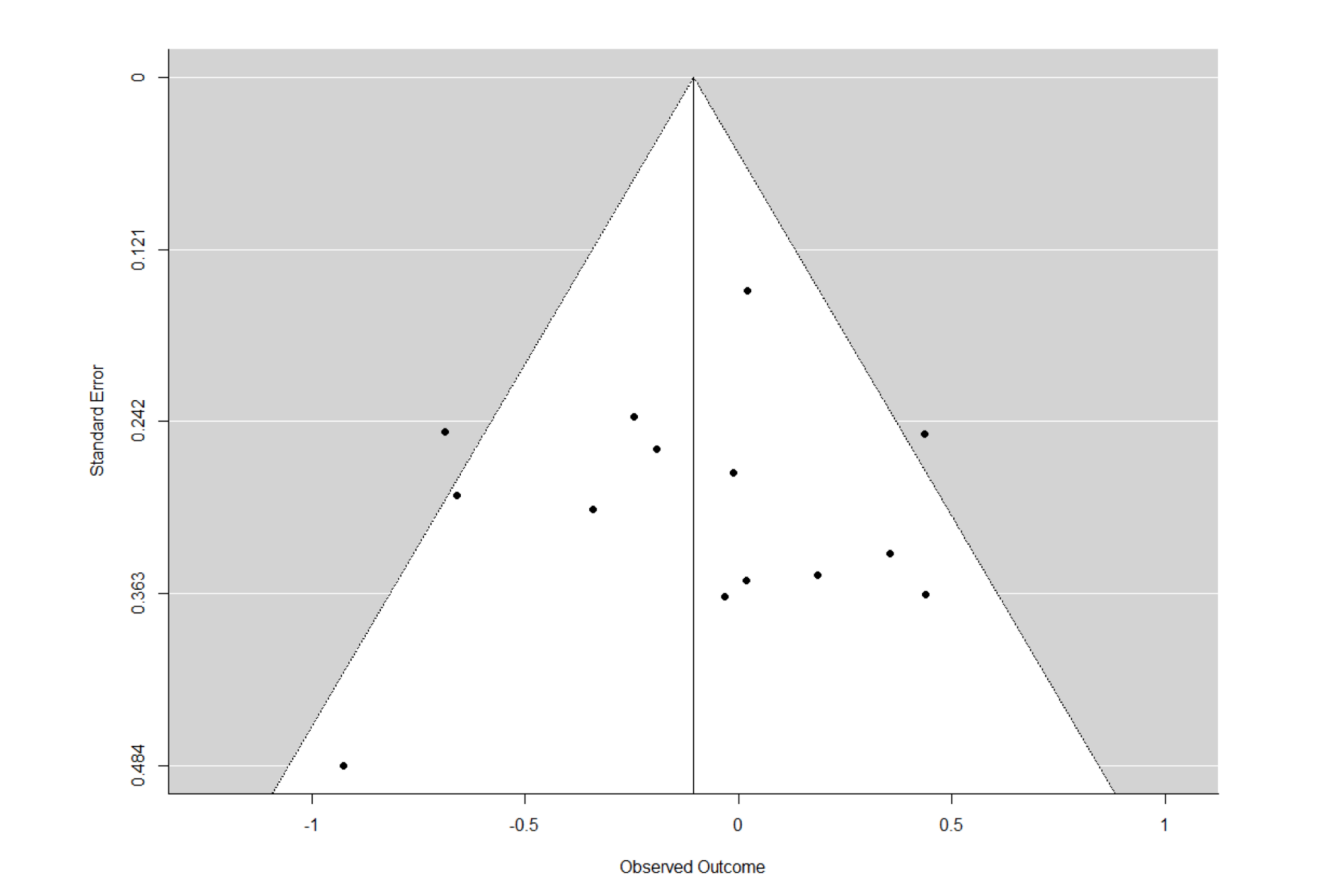
Funnel plots of standard errors plotted against effect sizes for identification of publication bias for cravings.

The Q statistics indicated that there was significant heterogeneity in the REMs of all studies and studies with psychotherapy for relapse. Furthermore, there was also significant heterogeneity in the MEMs for relapse, and cravings. The trim and fill analyses carried out for all REMs of all studies did not result in any imputation of studies. This suggests that publication biases were minimal or non-significant. The funnel plots and leave-one-out analyses are presented in Figures 6-9 and Tables 4-7. In general, the exclusion of any single study from the REMs did not alter the statistical significance of any existing pooled estimates; these estimates remained statistically significant in the REMs for abstinence, relapse, and GGT, and non-significant in the REM for cravings. These results suggest that the results are generally robust.

**Table 4:** Leave-one-out sensitivity analysis for abstinence. C = Coping; ST = Supportive Therapy.

	Study/Year	Estimate	P­_Estimate_	95% CI		Q	P_Q_
1	O'Malley et al. [5] (C)(1992)	0.31	0.0227	0.04	0.57	25.22	0.0471
2	O'Malley et al. [5] (ST) (1992)	0.24	0.0331	0.02	0.46	18.82	0.2220
3	Oslin et al. [6] (1997)	0.31	0.0209	0.05	0.58	25.26	0.0465
4	Volpicelli et al. [8] (1997)	0.31	0.0280	0.03	0.59	25.21	0.0472
5	Anton et al. [4] (2003)	0.29	0.0380	0.02	0.56	24.56	0.0562
6	Kranzler et al. [10] (2000)	0.35	0.0076	0.09	0.61	23.18	0.0803
7	Chick et al. [24] (2000)	0.34	0.0134	0.07	0.62	24.37	0.0591
8	Morris et al. [27] (2001)	0.29	0.0275	0.03	0.55	24.62	0.0552
9	Gastpar et al. [26] (2002)	0.34	0.0201	0.05	0.62	24.92	0.0511
10	Kiefer et al. [21] (2003)	0.25	0.0326	0.02	0.48	19.63	0.1867
11	Kranzler et al. [17] (2004)	0.27	0.0449	0.01	0.53	23.81	0.0683
12	Petrakis et al. [7] (2005)	0.34	0.0137	0.07	0.62	24.39	0.0588
13	Oslin [14] (2005)	0.35	0.0068	0.10	0.6	22.76	0.0893
14	Morley et al. [22] (2006)	0.33	0.0149	0.07	0.6	24.71	0.0539
15	O'Malley et al. [13] (2008)	0.26	0.0325	0.02	0.5	22.11	0.1048
16	Baltieri et al. [19] (2008)	0.33	0.0173	0.06	0.61	24.93	0.0509
17	Pettinati et al. [18] (2010)	0.34	0.0135	0.07	0.6	24.51	0.0570

**Table 5:** Leave-one-out sensitivity analysis for relapse. C = Coping; ST = Supportive Therapy; CBI = Combined Behavioral Intervention; NP = No psychotherapy; CBT = Cognitive Behavioral Therapy; MET = Motivational Enhancement Therapy.

	Study	Estimate	P­_Estimate_	95% CI		Q	P_Q_
1	O'Malley et al. [5] (C)(1992)	-0.44	<.0001	-0.59	-0.29	33.74	0.1417
2	O'Malley et al. [5] (ST) (1992)	-0.44	<.0001	-0.58	-0.29	35.76	0.0961
3	Anton et al. [3] (CBI) (2006)	-0.44	<.0001	-0.59	-0.28	41.27	0.0292
4	Anton et al. [3] (NP) (2006)	-0.42	<.0001	-0.57	-0.27	41.95	0.0248
5	Oslin et al. [6] (1997)	-0.41	<.0001	-0.56	-0.26	41.01	0.0310
6	Volpicelli et al. [8] (1997)	-0.41	<.0001	-0.56	-0.26	41.42	0.0282
7	Anton et al. [4] (2003)	-0.39	<.0001	-0.54	-0.24	40.09	0.0382
8	Chick et al. [24] (2000)	-0.41	<.0001	-0.56	-0.26	41.96	0.0248
9	Krystal et al. [30] (2001)	-0.41	<.0001	-0.58	-0.25	41.97	0.0247
10	Heinala et al. [25] (C)(2001)	-0.43	<.0001	-0.58	-0.28	35.02	0.1111
11	Heinala et al. [25] (ST) (2001)	-0.42	<.0001	-0.56	-0.27	41.94	0.0249
12	Morris et al. [27] (2001)	-0.41	<.0001	-0.56	-0.26	41.94	0.0249
13	Guardia et al. [20] (2002)	-0.40	<.0001	-0.55	-0.25	40.29	0.0366
14	Gastpar et al. [26] (2002)	-0.44	<.0001	-0.59	-0.29	40.13	0.0379
15	Latt et al. [28] (2002)	-0.40	<.0001	-0.55	-0.25	41.09	0.0304
16	Kiefer et al. [21] (2003)	-0.40	<.0001	-0.55	-0.25	39.93	0.0396
17	Balldin et al. [23] (2003) (CBT)	-0.41	<.0001	-0.56	-0.27	41.81	0.0257
18	Balldin et al. [23] (2003) (ST)	-0.42	<.0001	-0.57	-0.28	40.26	0.0368
19	Kranzler et al. [17] (2004)	-0.41	<.0001	-0.56	-0.26	41.79	0.0258
20	Ahmadi et al. [1] (2004)	-0.40	<.0001	-0.55	-0.25	40.78	0.0326
21	Oslin [14] (2005)	-0.43	<.0001	-0.58	-0.28	40.76	0.0328
22	Anton et al. [2] (2005) (CBT)	-0.40	<.0001	-0.55	-0.25	40.76	0.0328
23	Anton et al. [2] (2005) (MET)	-0.41	<.0001	-0.56	-0.26	41.94	0.0249
24	Morley et al. [22] (2006)	-0.44	<.0001	-0.59	-0.29	40.07	0.0384
25	O'Malley et al. [13] (2008)	-0.41	<.0001	-0.56	-0.26	41.15	0.0299
26	Toneatto et al. [29] (2009)	-0.41	<.0001	-0.56	-0.26	40.33	0.0362
27	Pettinati et al. [18] (2010)	-0.42	<.0001	-0.57	-0.28	41.56	0.0272
28	Monti et al. [11] (2001)	-0.42	<.0001	-0.57	-0.27	41.89	0.0252

**Table 6:** Leave-one-out sensitivity analysis for Gamma-Glutamyl Transferase. PCM = Primary Care Management; CBT = Cognitive Behavioral Therapy.

	Study/Year	Estimate	P­_Estimate_	95% CI		Q	P_Q_
1	Anton et al. [4] (2003)	-0.16	0.0203	-0.29	-0.02	3.74	0.9769
2	Petrakis et al. [7] (2005)	-0.16	0.0221	-0.29	-0.02	3.71	0.9777
3	Volpicelli et al. [8] (1997)	-0.15	0.0229	-0.28	-0.02	3.60	0.9802
4	O'Malley et al. [12] (2003) (PCM)	-0.15	0.0243	-0.28	-0.02	2.94	0.9915
5	O'Malley et al. [12] (2003) (CBT)	-0.17	0.0088	-0.30	-0.04	3.14	0.9887
6	O'Malley et al. [13] (2008)	-0.17	0.0091	-0.30	-0.04	3.24	0.9871
7	Brown et al. [16] (2009)	-0.16	0.0125	-0.29	-0.03	3.71	0.9776
8	Baltieri et al. [19] (2008)	-0.15	0.0228	-0.28	-0.02	3.62	0.9798
9	Kiefer et al. [21] (2003)	-0.16	0.0170	-0.29	-0.03	3.73	0.9772
10	Balldin et al. [23] (2003) (CBT)	-0.16	0.0125	-0.29	-0.04	3.69	0.9781
11	Balldin et al. [23] (2003) (ST)	-0.15	0.0231	-0.28	-0.02	3.27	0.9867
12	Morris et al. [27] (2001)	-0.18	0.0065	-0.32	-0.05	2.59	0.9951
13	Latt et al. [28] (2002)	-0.15	0.0207	-0.29	-0.02	3.68	0.9783

**Table 7:** Leave-one-out sensitivity analysis for cravings. PCM = Primary Care Management; CBT = Cognitive Behavioral Therapy.

	Study/Year	Estimate	P­_Estimate_	95% CI		Q	P_Q_
1	Anton et al. [4] (2003)	-0.09	0.4124	-0.31	0.13	22.94	0.0282
2	Davison et al. [9] (2004)	-0.14	0.1903	-0.34	0.07	21.14	0.0484
3	Kranzler et al. [10] (2000)	-0.05	0.6108	-0.23	0.13	17.18	0.1430
4	O'Malley et al. [12] (2003) (PCM)	-0.11	0.3018	-0.33	0.10	23.28	0.0254
5	O'Malley et al. [12] (2003) (CBT)	-0.12	0.2539	-0.34	0.09	22.72	0.0302
6	O'Malley et al. [13] (2008)	-0.09	0.4202	-0.30	0.13	22.70	0.0304
7	Brown et al. [16] (2009)	-0.08	0.4584	-0.28	0.12	20.37	0.0603
8	Baltieri et al. [19] (2008)	-0.11	0.3152	-0.32	0.10	23.36	0.0249
9	Guardia et al. [20] (2002)	-0.12	0.2898	-0.35	0.10	22.61	0.0312
10	Kiefer et al. [21] (2003)	-0.11	0.3106	-0.33	0.11	23.29	0.0253
11	Morley et al. [22] (2006)	-0.15	0.1115	-0.35	0.04	18.49	0.1016
12	Balldin et al. [23] (2003) (CBT)	-0.06	0.5330	-0.26	0.13	19.46	0.0779
13	Balldin et al. [23] (2003) (ST)	-0.10	0.3821	-0.32	0.12	23.24	0.0258
14	Latt et al. [28] (2002)	-0.13	0.1999	-0.34	0.07	21.49	0.0437

## Discussion

The current study examined the hypothesis that naltrexone is efficacious in treating AUD and psychotherapy on top of naltrexone significantly augments AUD-related treatment outcomes. In relation to former, our results have generally indicated that naltrexone has been efficacious in treating AUD. Specifically, the pool estimates indicated significant treatment effects in improving self-reported alcohol consumption outcomes such as abstinence, relapse as well as the objectively measured GGT, which is a widely used and highly specific biomarker for excessive alcohol consumption. However, there was not a significant treatment effect on cravings. In relation to the second hypothesis, adding psychotherapy to the naltrexone treatment of AUD did not significantly augment treatment outcomes; the combined treatment of psychotherapy and naltrexone was not significantly better than naltrexone alone.

The non-significant difference in treatment outcomes between studies with and without psychotherapy is noteworthy considering that previous meta-analytic research had found combined treatments significantly more effective than pharmacological treatment alone in treating mood and anxiety disorders. Nevertheless, while previous meta-analyses have noted significant and large treatment effects associated with psychotherapy among these affective disorders, psychotherapy has been less successful with AUDs. Meta-analyses have generally reported small and non-significant treatment effects associated with psychotherapy on AUD. Given these weak effects, one would not expect the addition of psychotherapy to significantly augment the naltrexone treatment of AUD. Indeed, researchers who investigated such a hypothesis within-study did not find significant differences between combined treatment and naltrexone-only treatment in influencing subsequent AUD outcomes [2, 3]. One may be concerned at the large variety of psychotherapies carried out across studies, thus rendering it difficult to interpret or generalize these results to the different adjunctive psychotherapies. This may be the case for the meta-analyzed relapse outcome, which was associated with significant heterogeneity. Notwithstanding the slight variation in the definition of relapse across studies, it may be possible that certain therapies when combined with naltrexone yield much better treatment outcomes than others. Nevertheless, despite the wide variety of psychotherapies carried out across the different studies, the results among studies with psychotherapy were not significantly heterogeneous, at least in relation to abstinence, GGT and cravings. Assuming the naltrexone effect is constant across study, this may hint to the dodo bird verdict – all psychotherapies, regardless of their theoretical orientations, produced similar outcomes. Regardless, future researchers may consider comparing treatment outcomes between various psychotherapies in combination with naltrexone to verify such an interpretation.

It is also interesting that while naltrexone had resulted in significantly improved treatment outcomes in terms of self-reported measures and biomarkers of alcohol consumption, it did not seem to have a significant treatment effect in reducing alcohol-related cravings. There may be two explanations for this. First, while naltrexone reportedly modulates the dopaminergic activity in the mid-brain reward system in an attempt to inhibit the reinforcement associated with alcohol consumption, it does little to alleviate alcohol-related cravings which are largely associated with activity in the prefrontal and limbic regions of the brain. Second, it is plausible that alcohol-related cravings will decrease on its own even without any naltrexone treatment and this decrease will therefore mask the naltrexone treatment effect, if any at all on cravings. Indeed, we observed that several studies reported significant decreases (with respect to baseline) in cravings among placebo groups which were comparable to those of the naltrexone treatment groups. Furthermore, this decrease cannot be explained by the adjunctive psychotherapy in most of the included studies since it was not a significant moderator in the MEM; the pooled estimates relating to the decrease in cravings were also similar between studies with and without psychotherapy.

These findings present a key implication in the treatment of AUD. These results suggest that it is not necessary for adjunctive psychotherapy to be carried out on top of naltrexone in the treatment of AUD. From a resource-allocation perspective, given that individual psychotherapy is a relatively time- and resource-consuming process, the resources associated with such adjunctive psychotherapies can be conserved, or instead should be directed at other clinical populations, such as those with depression and anxiety disorders, in which psychotherapy would be a lot more beneficial.

The current results are subjected to three major limitations. Firstly, given the very limited number of studies on naltrexone treatment without psychotherapy, their pooled estimates may not reliably reflect the effect of naltrexone treatment alone. More ‘naltrexone-only’ intervention studies should be carried out such that future meta-analyses can robustly compare the AUD treatment outcomes between combined-intervention and naltrexone-only treatments. Secondly, it is possible that between-study differences in methodology or participants’ characteristics among the included studies may confound the inclusion of psychotherapy in influencing treatment outcomes. Future intervention studies on AUD should be carried out to examine the inclusion of psychotherapy on top of naltrexone treatment within-study, such that stronger conclusions relating to the addition of psychotherapy on top of naltrexone can be made. Thirdly, the manner in which the additive effects of psychotherapy were studied in the current meta-analysis was less than optimal, given that we have simply compared studies with or without psychotherapy, instead of studies with psychotherapy or a control condition for psychotherapy. Furthermore, unlike the naltrexone condition, the psychotherapy conditions were not randomly assigned across all participants. This might raise concerns relating to whether psychotherapy effects were genuine or attributable to psychotherapy-placebo effects. Finally, as a result of our study inclusion criteria, such as English language studies only and including outcomes studied by three studies without therapy, we excluded a large number of studies, including one with a large sample. This creates a selection bias and may raise concerns relating to the generalizability of the results.

## Conclusions

Naltrexone treatment is efficacious in reducing alcohol consumption, but not reducing cravings. Adding psychotherapy on top naltrexone did not result in any significant additional benefit for AUD patients.
